# Metabolomics Analysis Reveals the Potential Advantage of Artificial Diet-Fed *Bombyx Batryticatus* in Disease Treatment

**DOI:** 10.3390/metabo16010051

**Published:** 2026-01-07

**Authors:** Han Chen, Yuting Feng, Daorui Pang, Qiong Yang, Yuxiao Zou, Ping Lin, Guanwang Shen, Dongxu Xing

**Affiliations:** 1 Sericultural & Agri-Food Research Institute, Guangdong Academy of Agricultural Sciences: Key Laboratory of Functional Foods, Ministry of Agriculture and Rural Affairs/Guangdong Key Laboratory of Agricultural Products Processing, Guangzhou 510610, China; ch1737767073@163.com (H.C.); daorui66@163.com (D.P.); serilover@126.com (Q.Y.); yuxiao_zou@126.com (Y.Z.); 2 Integrative Science Center of Germplasm Creation in Western China (Chongqing) Science City, Biological Science Research Center, Southwest University, Chongqing 400716, China; fqwertyuiop@email.swu.edu.cn (Y.F.); linpingswu@swu.edu.cn (P.L.)

**Keywords:** *Bombyx Batryticatus*, UPLC-MS/MS, metabolomics, isoflavonoid biosynthesis, artificial diet, disease

## Abstract

**Background:** *Beauveria bassiana* infection of silkworm forms *Bombyx Batryticatus* (BB). It is a medicinal material with significant pharmacological potential. While artificial diet feeding improves the production efficiency of BB, it might alter host metabolism, consequently affecting its bioactive components and efficacy. To address this, we conducted a metabolomics analysis of BB reared under different feeding conditions; **Methods:** UPLC-MS/MS was employed to conduct metabolomic analysis of BB under three rearing conditions: all instars mulberry leaf feeding (MF), all instars artificial diet feeding (AF), and mixed feeding (AMF). The sample collection time was selected as the time when silkworms died after infection (D0), and the fifth day after death (D5), which is the time when fungus produces biologically active secondary metabolites to reach a stable state; **Results:** Compared to MF, AF did not significantly alter the levels of the index component induced by *B. bassiana* infection—beauvericin. Moreover, the overall metabolic profile differences between the two groups decreased at the later stage (D5). Specifically, the average Pearson correlation between these groups was 0.659 ± 0.102, and the first two principal components of PCA explained 49.6% of the total variance. This suggests a reduction in the differences in their pharmacological active components. KEGG enrichment analysis revealed that AF promoted the accumulation of certain flavonoids (e.g., apigenin, luteolin), but, overall, the biosynthesis of flavone and flavonol is suppressed. Additionally, several metabolites, including N,N′-diferuloylputrescine, N-methyl-4-aminobutyric acid, and 3,4-dimethoxyphenylacetic acid, were identified to be significantly positively correlated with artificial diet supplementation; **Conclusions:** This study reveals metabolic differences in BB under different rearing methods at the metabolomic level, providing a scientific basis for evaluating the quality of this medicinal material.

## 1. Introduction

*Beauveria bassiana* is a globally distributed entomopathogenic fungus with a broad host range. It holds significant research value in the fields of biological control [1,2] and medicinal applications [3]. In medicine, when fourth- or fifth-instar silkworm larvae are infected and killed by this fungus, their dried carcasses form a medicinal product known as *Bombyx Batryticatus* (BB) [4]. As a traditional Chinese medicinal material, it has long been used to treat various ailments. Its applications include calming endogenous wind, dispelling pathogenic wind for relieving pain, dissipating phlegm, and resolving masses [4,5]. Modern medical research has further revealed that BB possesses significant biological activities, including antitumor, anticoagulant, antimicrobial, and antioxidant effects [6]. Studies have confirmed that water extracts [7], ethanol extracts [8], and crude protein extracts [9] of BB exhibit notable bioactivity. This promotes neuronal cell repair [10], inhibits the proliferation of liver cancer cells, induces apoptosis in gastric cancer cells [9], and exerts anti-inflammatory effects—underscoring its considerable medicinal potential. Furthermore, compounds such as beauvericin have been identified as key bioactive constituents of BB. For instance, beauvericin present in BB can significantly inhibit the growth, clonogenicity, migration, and invasion of A375SM human melanoma cells [11]. The oligosaccharide BBPW-2 isolated from BB extracts suppresses the activity of cervical cancer (HeLa), liver cancer (HepG2), and breast cancer (MCF-7) cells [12]. Nevertheless, the specific bioactive compounds responsible for these effects remain poorly characterized. This substantially hinders the clinical application and industrial development of BB.

Currently, BB is primarily obtained by infecting mulberry leaf-reared silkworms with *B. bassiana*. However, as oligophagous insects, silkworms can only feed on Moraceae plants (such as mulberry leaves or *Cudrania tricuspidata* leaves) [13]. Their large-scale production is constrained by seasonal variations and labor costs. As a result, artificial diet for silkworms has been developed. Research on silkworm artificial diet has been conducted for over five decades [14], and the technology is now relatively mature [15]. Currently, mainstream diets mainly consist of mulberry leaf powder, corn powder, defatted soybean powder, cellulose, molding agents, vitamins, preservatives, and inorganic salts [16]. Despite this technological advancement, previous studies have indicated that changes in dietary composition can significantly alter the metabolic profiles of silkworm hemolymph, midgut, and other tissues [17,18], and also modulate their physiological condition, cocoon quality, and gut microbiota [13,14,19]. This raises the question: have the pharmacologically active substances in artificial diet-fed BB changed compared with those in mulberry leaf-fed BB?

Given the diverse pharmacological functions and the complexity of bioactive substances in BB, in the present study, we employed ultra-performance liquid chromatography–tandem mass spectrometry (UPLC-MS/MS)-based metabolomics. Our aim was to reveal metabolite differences between artificial diet feeding in all instars (AF), mulberry leaf feeding in all instars (MF), and artificial diet feeding in the first three instars and mulberry leaf feeding in the fourth and fifth instars (AMF). These findings will provide new insights into the pharmacological properties and characteristics of artificially produced BB.

## 2. Materials and Methods

### 2.1. Silkworms Rearing and B. bassiana Infection

Silkworms and *B. bassiana* were provided by the Sericulture and Agri-Food Research Institute of the Guangdong Academy of Agricultural Sciences (Guangzhou, China). The silkworms were divided into three groups according to different feeding patterns: artificial diet feeding in all instars (AF), mulberry leaf feeding in all instars (MF), and artificial diet feeding in the first three instars and mulberry leaf feeding in the fourth and fifth instars (AMF). Three biological replicates were used for each treatment group. All experimental groups were housed under identical environmental conditions, maintained at a temperature of 25 ± 0.5 °C, a relative humidity of 90 ± 5%, and a 12 h light/dark cycle throughout the rearing period. *B. bassiana* was inoculated into the fifth instar silkworms, as previously described [4].

### 2.2. Samples Preparation and Metabolite Extraction

Infected and stiff silkworms were collected on Day 0 (D0, recently deceased, respectively named AF1, MF1, and AMF1) and Day 5 (D5, respectively named AF2, MF2, and AMF2). It is worth noting that the collected samples represent a complete combination of host insect tissue, the fungus infected with *Beauveria bassiana*, and any undigested dietary substances present in the intestinal tract. Therefore, whole-body metabolomics analysis captured metabolites derived from all these components. Samples were freeze-dried using a vacuum freeze dryer (Scientz-100F; Scientz, Ningbo, China) and crushed using a mixer mill (MM400; Retsch, Haan, Germany) with zirconia bead for 1.5 min at 30 Hz. Next, 100 mg of the lyophilized powder was dissolved in 1.2 mL of 70% methanol solution. The mixture was vortexed for 6 cycles (30 s per cycle with 30min intervals between cycles) to ensure complete metabolite extraction. Finally, the samples were stored overnight at 4 °C and centrifuged at 12,000 rpm for 10 min to obtain supernatants. The extract was filtered through an organic-phase nylon microporous membrane (0.22 μm) before UPLC-MS/MS analysis.

### 2.3. UPLC-MS/MS

Metabolite data were collected using an ultra-performance liquid chromatography system (Nexera X2, SHIMADZU, Kyoto, Japan) coupled with a tandem mass spectrometry system (4500 QTRAP, Applied Biosystems, Waltham, MA, USA). Chromatographic separation was performed on an Agilent SB-C18 column (1.8 µm, 2.1 mm × 100 mm) with mobile phase A (ultrapure water containing 0.1% formic acid) and mobile phase B (acetonitrile with 0.1% formic acid) at a flow rate of 0.35 mL/min. The column temperature was maintained at 40 °C, and 4 μL of sample was injected per analysis. The effluent was directed to an electrospray ionization (ESI)-triple quadrupole-linear ion trap mass spectrometer (AB4500 QTRAP UPLC/MS/MS, Hamburg, Germany) for detection. The ion source temperature was set to 550 °C with turbo spray interface, and the ion spray voltage was 5500 V (positive ion mode) or −4500 V (negative ion mode). Ion source gas I (GSI), gas II (GSII), and curtain gas (CUR) were set to 50, 60, and 25 psi, respectively, and the system was operated with collision-induced ionization under high-sensitivity parameters.

### 2.4. Processing and Statistical Analysis of Metabolomics Data

#### 2.4.1. Raw Data Processing and Quality Control

The spectrometry data were processed using Analyst software (version 1.6.3). Metabolites were identified based on MS/MS spectral matching against the MetWare database (MWDB; MetWare Biotechnology Co., Ltd., Wuhan, China). During data processing, potential interference peaks, such as isotopic peaks, common adduct ions (e.g., K^+^, Na^+^, NH_4_^+^), and fragment ions derived from in-source dissociation of larger molecules, were removed to ensure the accuracy of metabolite identification and quantification. Metabolite quantification was performed using multiple reaction monitoring (MRM). MultiQuant software was used for chromatographic peak correction, and the peak area was calculated to represent the relative content of each corresponding metabolite.

#### 2.4.2. Principal Component Analysis (PCA) and Partial Least Squares Discriminant Analysis (PLS-DA)

The data were unit variance scaled, and an unsupervised PCA was performed using the “prcomp” statistical function in R (version 3.5.1). The variance of each principal component was calculated to evaluate the model’s representativeness. Partial least squares discriminant analysis (PLS-DA) was used to validate the PCA model and determine the variable importance in projection (VIP) values for each metabolite.

#### 2.4.3. Hierarchical Cluster Analysis and Pearson Correlation Coefficients

The R package ComplexHeatmap (version 2.8.0) was used to perform hierarchical clustering of samples and metabolites and to generate heatmap visualizations. Pearson correlation coefficients between samples were calculated using the Hmisc package (version 4.4.0) in R.

#### 2.4.4. Differential Metabolite Identification

Significantly regulated metabolites between groups were identified by VIP ≥ 1 and absolute log_2_FC (fold change) ≥ 1 (AF2 vs. AF1 were made by calculating the ratio of AF2 to AF1, with other comparisons performed similarly). In the univariate analysis, the VIP values were extracted from the OPLS-DA results, which also contained score and permutation plots and were generated using the R package MetaboAnalystR (1.0.1) to preliminarily screen the differential metabolites (DMs) between the groups, and the log_2_FC value was used for further DMs identification. The metabolites were classified into three levels according to the score of the secondary mass spectrometry results and the mapping score between the retention time and reference database to filter the most potential DMs: level 1: The secondary mass spectrometry, retention time (RT) of the sample substance matches the database substance with a score of 0.7 or above, which is the highest confidence level of the identification result; level 2: The secondary mass spectrometry, RT and database substance matching scores of the sample substances range from 0.5 to 0.7 points, which are relatively reliable presumptive annotation results; and level 3: The mass-charge ratio (Q1, Q3), RT, Declustering Potential (DP), and Collision Energy (CE) of the sample substances were consistent with those of the database substances, which is a preliminary structural presumption.

#### 2.4.5. KEGG Annotation and Enrichment Analysis

The identified metabolites were annotated using the KEGG compound database “http://www.kegg.jp/kegg/compound/ (accessed on 20 August 2023)”. Annotated metabolites were mapped to the KEGG Pathway database “http://www.kegg.jp/kegg/pathway.html (accessed on 20 August 2023)”. Thereafter, path-ways with significantly regulated metabolites were subjected to metabolite set enrichment analysis (MSEA) and their significance was determined using hypergeometric test *p*-values. A differential abundance (DA) score was calculated to evaluate the cumulative changes in all differential metabolites (DMs) within each pathway.

## 3. Results

### 3.1. Quality Control and Variance Statistics

**Figure 1:**
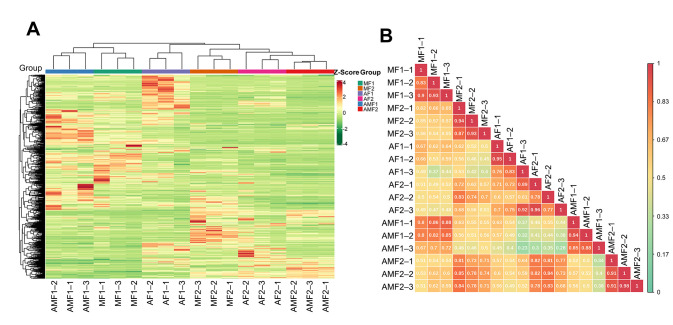
Metabolites analysis and sample statistics. (**A**) Heat maps of the 822 identified metabolites; (**B**) Pearson correlation analysis of samples.

A total of 822 metabolites (Figure 1A) were identified under predetermined experimental conditions in our metabolomic analysis of all samples. These metabolites were primarily classified into 10 categories (Figure S1A,B), including 214 level 1 metabolites (score > 0.7), 80 level 2 metabolites (0.5 < score ≤ 0.7), and 528 level 3 metabolites (score < 0.5). To evaluate analytical reliability, we performed overlay analysis of total ion chromatograms (TICs) from QC samples (Figure S1C). The experimental data showed high stability, evidenced by highly consistent retention times and peak intensities; quantitatively, this was supported by over 75% of metabolites in the QC samples having an RSD of less than 30% (Figure S1D). This validation ensured the reliability of subsequent data analyses. The Pearson correlation coefficient was calculated to analyze the correlation between samples (Figure 1B), and PCA was used to analyze the similarity between samples (Figure S2). We conducted PERMANOVA analysis on the principal component scores (PC1: 30.03% and PC2: 19.56%) from the metabolic profile. The results indicated significant overall differences among the AF1, MF1, and AMF1 groups (pseudo-F = 31.74, *p* = 0.008). Pairwise comparisons confirmed that the MF1 and AMF1 groups had high similarity and correlation (pseudo-F = 7.42, *p* = 0.028, bonferroni-adjusted *p* = 0.084), but the similarity between the AF1 group and AMF1 groups (pseudo-F = 29.63, *p* = 0.008, bonferroni-adjusted *p* = 0.024) or MF1 groups (pseudo-F = 31.74, *p* = 0.008, bonferroni-adjusted *p* = 0.024) was poor. For D5, PERMANOVA showed statistically significant overall differences among the AF2, MF2, and AMF2 groups (pseudo-F = 31.7, *p* = 0.008). However, from the heat map, the differences among the three groups narrowed. Specifically, at D5, the MF2 and AF2 groups showed metabolic similarity, with an average Pearson correlation of 0.66.

### 3.2. Effects of Artificial Diet Feeding on the Stiff Stage of BB

**Figure 2:**
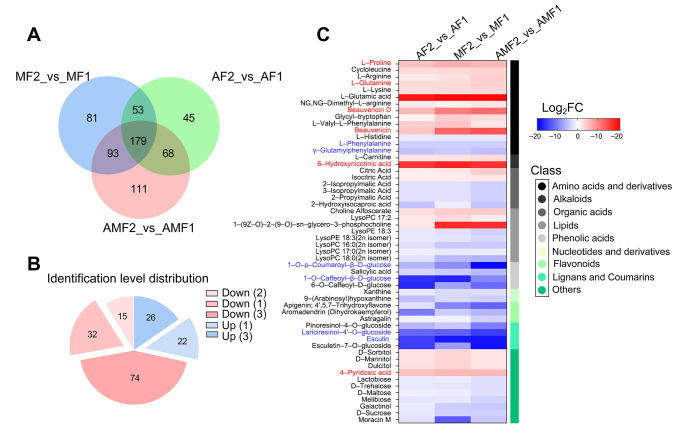
DMs filtering results in three groups. (**A**) Venn diagrams of three groups with different changing trends; (**B**) Metabolite statistics of the three groups with consistent changing trends, the numbers in parentheses indicate the corresponding map level; (**C**) Heatmap of 54 DMs with similar expression trends.

The formation of BB is characterized by dynamic alterations in marker metabolite profiles, which serve as critical biochemical indicators of the stiff stage [4]. To evaluate whether artificial diet-fed BB affects the stiff stage, we first compared the DMs among the three groups. Consequently, 346, 451, and 406 DMs were obtained in AF1_ vs. _AF2, AMF1_ vs. _AMF2, and MF1_ vs. _MF2, respectively (Figure S3A–C), indicating that there were 179 common DMs in the three comparisons (Figure 2A). Notably, we also found that 169 common DMs met the same change trend during the stiffening stage, of which 48 DMs accordantly increased and 121 DMs accordantly decreased in the three comparison groups (Figure 2B). We next conducted relative content statistics on 54 DMs at level 1 and found that many DMs, including L-proline, L-glutamic acid, beauvericin D, beauvericin, 6-hydroxynicotinic acid, and 4-pyridoxic, were upregulated during the stiffening stage. In addition, many DMs, such as L-phenylalanine, γ-glutamylphenylalanine, 1-O-p-coumaroyl-β-D-glucose, 1-O-caffeoyl-β-D-glucose, lariciresinol-4′-O-glucoside, and esculin (6,7-dihydroxycoumarin-6-glucoside), were downregulated during the stiffening stage (Figure 2C, Table S2). Beauvericin has been suggested as an indicator metabolite of BB stiff stage [4]. Our results showed that artificial diet addition had no significant effect on beauvericin and beauvericin D during BB production.

Comparing differential metabolites (DMs) between stiffening time points (D0 vs. D5) across the AF groups and other groups enables more intuitive prediction and analysis of therapeutic response variations in disease treatment. Since the correlation and PCA results showed that the AMF and MF group samples exhibited high similarity at D0(R ≈ 0.789 ± 0.075) (Figure 1B and Figure S2), we focused on DMs with the same changing trend in the AMF and MF groups but different trends in the AF group. The results showed that 128 DMs met this filtering condition, and most DMs were level 3 mapped results in the database (Figure S4). Among the 128 DMs screened out, only gallic acid and lysoPE 18:4 showed complete direction reversals (Table S3-1). The vast majority of DMs (126 DMs) had similar content change trends during the stiffening process between the AF group and the MF/AMF group. Differences were more reflected in the amplitude or significance of the changes. This indicates that after silkworms are infected with *Beauveria bassiana*, their core metabolic stress and response programs are highly conserved under different nutritional backgrounds. We sorted the DMs with a matching level of 1 for further screening of the AF-specific metabolites. Among the obtained 27 DMs, 15 were found to have significant changes in AMF and MF but did not change significantly in the AF group during the stiffening stage (Table 1). Furthermore, only 3-O-methylgallic acid was upregulated, while the other 14 DMs, including kaempferol-3-O-galactoside (trifolin), were significantly downregulated. Comparatively, there were 12 DMs that showed significant differences before and after stiffening in the AF group, whereas there was no difference between the AMF and MF groups (Table 2). Three flavonoids, kaempferol (3,5,7,4′-tetrahydroxyflavone), morin, and quercetin, which are significantly downregulated during the stiffening stage, are particularly noteworthy.

### 3.3. KEGG Enrichment Analysis of DMs

**Figure 3:**
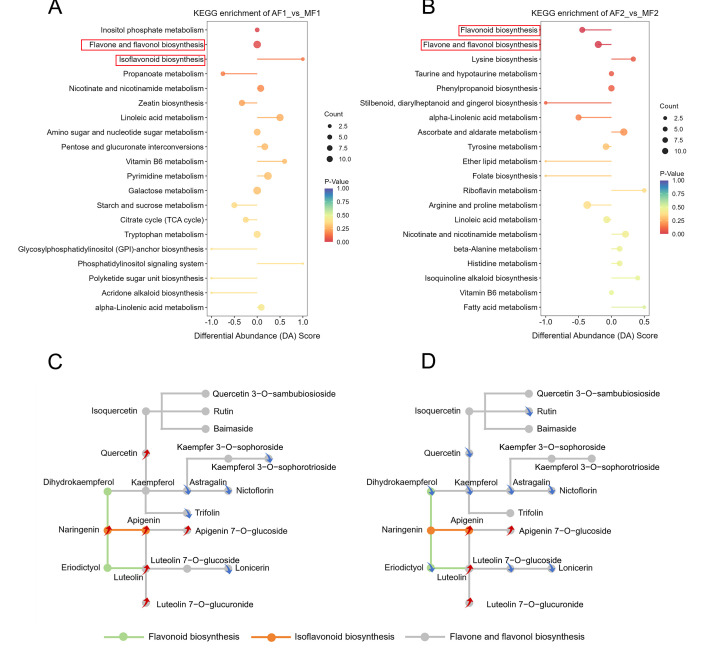
KEGG enrichment and relationship of DMs between groups. (**A**,**B**) indicate the KEGG enrichment of DMs in the AF and MF groups on D0 and D5, respectively; (**C**,**D**) Changes in flavonoids biosynthesis-related DMs in the AF and MF groups on D0 (**C**) and D5 (**D**) respectively (red arrows: up-regulation; blue arrows: down-regulation).

Our previous KEGG results showed that flavone metabolism-related pathways (flavone and flavonol biosynthesis, flavonoid biosynthesis, and isoflavonoid biosynthesis pathways) were especially enriched in the AF and AMF groups (Figure S3D,E). This implied that flavones may be the major DM in artificial diet-fed BB. Subsequently, we compared AF and MF at both stiff time points to analyze the DMs and KEGG enrichment. As expected, DMs were significantly enriched in flavone metabolism-related pathways (Figure 3A,B). Thereafter, we found that 19 DMs were enriched in three pathways that may be related to mutual transformation (Figure 3C,D). Although the apigenin (level 3), apigenin 7 O-β-D-glucoside (level 3), and luteolin 7 O-β-D-glucuronide (level 1) decreased in the AF group during stiffening (Figure S5A), the flavonoid-related metabolites (apigenin, apigenin 7 O-β-D-glucoside, luteolin (level 1), and luteolin 7 O-β-glucuronide) were upregulated in the AF group compared to the MF group at both D0 and D5 (Figure 3C,D). The levels of kaempferol (level 1) and its metabolic products, astragalin (level 1) and nictoflorin (level 1), were significantly lower in the AF group than in the MF group. Furthermore, we found that in the AMF and MF groups, with the development of stiffening, the metabolites kaempferol-3-O-galactoside (trifolin) (level 1) and quercetin-3-O-glucuronide (level 1) significantly decreased and increased, respectively, whereas no such change was observed in the AF group (Figure S5B,C). This evidence indicates that artificial diet-fed BB, which is different from feeding mulberry leaves, promotes the biosynthesis of isoflavonoid on D0, while depressing flavonoid biosynthesis and flavone and flavonol biosynthesis during the stiffening stage.

### 3.4. Analysis of Metabolites Related to Artificial Diet Addition

BB contains diverse bioactive compounds, including but not limited to proteins, peptides, fatty acids, flavonoids, nucleosides, steroids, coumarins, and polysaccharides [5]. Some of them are closely related to clinical efficacy, such as flavonoids and oxalic acid [4]. To preliminarily analyze the differences between the DMs in the AF group and those in MF and AMF groups, we first performed a comparison of metabolite differences between the groups at two stiffening time points (D0 and D5). We have respectively obtained 366, 337, and 267 DMs at D0, and 301, 309, and 326 DMs at D5 in three comparisons (Table 3). In order to screen the metabolites with best group representativeness, we conducted a common analysis of DMs in the three groups. A total of 77 and 60 common DMs were screened in D0’s and D5’s comparisons (Figure S6A,B), respectively. Statistics on mapping results show that these substances class into nine biological categories, and the heatmap for magnitude of the between-group differences is shown in Figure S6C,D.

**Figure 4:**
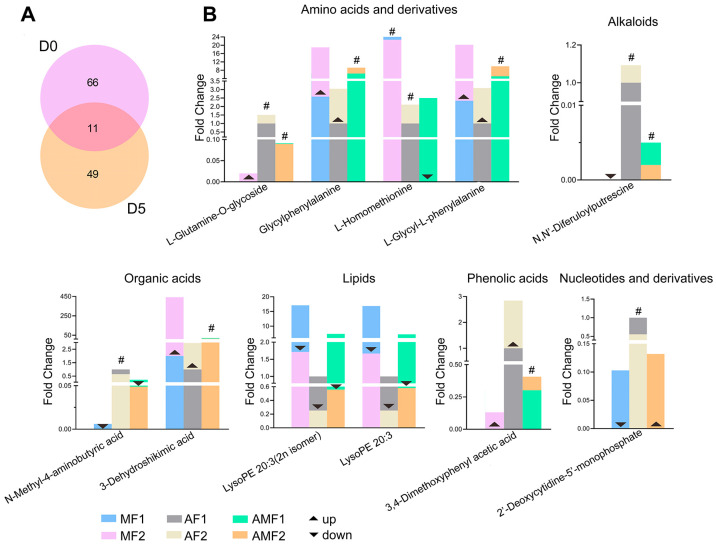
Analysis of DMs variation between the groups. (**A**) Venn diagram of the level 1 screening; (**B**) Changes in and differences in DMs, the symbol “#” indicates no significant difference; arrows show the regulation direction of differential metabolites in D5 versus D0 (▲: up-regulation, ▼: down-regulation).

Subsequently, we investigated whether specific metabolites exhibited significant correlations with artificial diet supplementation at both D0 and D5 time points, while analyzing their temporal dynamics. A total of 11 DMs (level 1) were identified and categorized into 6 biological substances (Figure 4A, Table S4). Considering that artificial diet feeding amount may be positively or negatively correlated with key metabolites, we focused on metabolites with progressively different contents in the AF, AMF, and MF groups. A total of 7 DMs (L-glutamine-O-glycoside, L-homomethionine, N,N’-diferuloylputrescine, N-methyl-4-aminobutyric acid, lysoPE 20:3(2n isomer), lysoPE 20:3, 3,4-dimethoxyphenyl acetic acid) met this differential condition setting at D0, and 9 DMs (glycylphenylalanine, L-glycyl-L-phenylalanine, N,N′-diferuloylputrescine, N-methyl-4-aminobutyric acid, 3-dehydroshikimic acid, lysoPE 20:3, lysoPE 20:3(2n isomer), 3,4-dimethoxyphenyl acetic acid, 2′-deoxycytidine-5′-monophosphate) met this differential condition setting at D5 (Figure 4B). Among them, N,N’-diferuloylputrescine, N-methyl-4-aminobutyric acid, and 3,4-dimethoxyphenylacetic acid, present at both D0 and D5, showed a positive correlation with artificial diet addition. Only N,N’-diferuloylputrescine was mapped at level 1 in our data annotations (Table S4).

## 4. Discussion

The period from the death of silkworms infected by *B. bassiana* (D0) to the formation of white spores on the larval surface (D5) represents a critical phase. This phase involves the formation and accumulation of pharmacologically active substances during the stiffening process. Consequently, it largely determines the final medicinal value of BB [4]. In this study, we employed Pearson correlation and principal component analyses. These analyses revealed that, during the late stiffening stage (D5), metabolites from different feeding groups (AF2, AMF2, and MF2) exhibited a certain degree of similarity. The similarity in metabolite composition provides preliminary chemical evidence. It suggests that BB obtained under both rearing methods may exhibit consistent medicinal efficacy. However, this hypothesis requires further validation through direct pharmacological or bioactivity analyses in future studies. Beauvericin, a key marker compound reflecting the degree of stiffening and bioactivity [4], accumulated significantly across all groups (Figure 2C). And its content was not substantially affected by the rearing method. This further demonstrates that artificial diet feeding does not alter the core stiffening process. It also does not change the composition of fundamental active substances in BB. These findings provide an important theoretical basis for the promotion and application of artificially reared BB. Nonetheless, potential differences in specific pharmacological effects require further validation through functional assays.

Previous studies have highlighted the importance of flavonoid metabolites in the formation of BB [4]. The current study revealed that various metabolites associated with flavonoid pathways were reduced to varying degrees in the AMF and AF groups (Figure S3). The isoflavonoid biosynthesis pathway was enhanced. These results suggest that the addition of artificial diet during the rearing process of BB is associated with alterations in flavonoid metabolism. Compared with the MF group, the AF group exhibited significantly higher levels of apigenin (level 3), luteolin (level 1), and their derivatives both at D0 and D5 (Figure 3C,D). Apigenin and luteolin are typical plant-based flavonoids that cannot be synthesized by animals. Apigenin is widely present in various plants, especially in the Asteraceae family. Additionally, it has multiple forms in the Fabaceae family, including glucosides, O-methyl ethers, C and O-glucosides, acetylated derivatives and their aglycone, etc. [20]. Luteolin and its glycosides (such as luteolin glycosides) have been identified in over 300 plant species. Within the Asteraceae, Lamiaceae, Poaceae, Leguminosae, and Scrophulariaceae families, LUT and its glycosides have been identified in 66, 38, 13, 10, and 10 species, respectively [21]. Our metabolome results showed that in the MF group, apigenin levels were at a low level on D0 and dropped below the detection limit on D5. In contrast, in the AF group, its initial level at D0 was significantly higher. Despite some consumption during the stiffening process, it ultimately remained at a relatively detectable high level. Luteolin was not detected in either D0 or D5 in the MF group. While in the AF group, its content remained stable and detectable throughout the stiffening process. This accumulation pattern is highly correlated with the plant composition of the diets. Although mulberry leaves contain flavonoids, apigenin and luteolin are not their dominant components. Artificial diet contains legumes such as soybean meal, which are known rich sources of apigenin and luteolin. Therefore, we hypothesize that these two flavonoids in the AF group originated from the legume components in the artificial diet and accumulated in the silkworms, thereby causing changes in the content of specific flavonoid active substances in the silkworm pupae. Existing studies have confirmed that apigenin possesses hypoglycemic, anti-inflammatory, and pro-apoptotic properties. It can also inhibit tumor growth by modulating the Bcl-2/Bax/STAT-3 signaling pathway [22]. Furthermore, substantial evidence suggests luteolin’s potential value in the treatment of neurological disorders and diabetes prevention [23,24]. Notably, luteolin-7-O-β-glucuronide (level 1) also increased significantly during the stiffening process in the AF group (Figure S5A). This compound has been demonstrated to play a role in regulating apoptosis in immortalized non-tumor human keratinocytes and Jurkat cells [25,26], which may represent a unique advantage of artificially reared BB.

Furthermore, the study identified multiple metabolites that showed significant correlations with artificial diet supplementation. Among these (level 1), the contents of N,N’-diferuloylputrescine, N-methyl-4-aminobutyric acid, and 3,4-dimethoxyphenyl acetic acid increased gradually with the increase in feed supplemental level. None of these three DMs are direct formula components of the artificial feed used in this study, but their sources and dynamic change patterns reveal potential formation pathways. N,N′-diferuloylputrescin is a common phenolic amine substance in plants, commonly found in plants such as corn [27]. N-methyl-4-aminobutyric acid is a methylated derivative of the neurotransmitter γ -aminobutyric acid that naturally exists in plants such as *Glycyrrhiza uralensis* Fisch [28]. In the MF groups, both N,N′-diferuloylputrescine and N-methyl-4-aminobutyric acid were low on D0 and continued to decrease during the stiffening process. On D5, they could no longer be detected. In contrast, both compounds maintained a relatively high level in the AF/AMF groups. This indicates that they are very likely derived from plant-based raw materials in artificial diet (such as soybean meal, corn flour, etc.) and accumulate as characteristic metabolites in silkworms. 3,4-dimethoxyphenyl acetic acid is a secondary metabolite derivative of plants/microorganisms, a derivative of the phenylpropanoid metabolic pathway, and present in plants such as *Artemisia scoparia* [29]). Its content showed a continuous upward trend in the stiff process of the MF/AMF/AF groups. Notably, on Day 0, it was detected only in groups fed artificial diet (AF/AMF) but not in the MF group; on Day 5, it also appeared in the MF group. This dynamic feature suggests that the source of 3,4-Dimethoxyphenyl acetic acid has a dual origin. Its initial accumulation may be related to the plant components in the feed. However, the general increase in content during the stiffening process is likely mainly due to the active phenylpropanoid metabolism during the growth of *Beauveria bassiana*.

Previous studies have reported that N,N’-diferuloylputrescine exhibits inhibitory activity against α-glucosidase [30]. More importantly, 1-deoxynojirimycin—another α-glucosidase inhibitor extracted from BB—has been demonstrated to exert protective effects in diabetic mice. It reduces total cholesterol, triglycerides, fasting blood glucose, and glycated hemoglobin levels [31]. N-Methyl-4-aminobutyric acid can be metabolized into γ-aminobutyric acid (GABA), a major inhibitory neurotransmitter in the central nervous system. Administration of GABA has been shown to induce relaxation and alleviate anxiety within one hour. It may also enhance immune function under stress conditions [32,33]. 3,4-Dimethoxyphenylacetic acid is a common intermediate in chemical synthesis. It is used in the production of β-blockers such as bevantolol and antiarrhythmic agents like verapamil. And it also serves as a key precursor in the synthesis of isoquinoline alkaloids [34]. These findings indicate that BB produced through artificial diet feeding may yield a broader spectrum of bioactive substances compared to those obtained from traditional mulberry leaf-based feeding.

## 5. Conclusions

BB obtained through *B. bassiana* infection of silkworm reared on an artificial diet retain their typical characteristics; however, the dietary shift significantly alters the flavone metabolism-related pathways in the infected hosts. Furthermore, due to the inclusion of non-mulberry components in the artificial diet, the resulting BB exhibit a more diverse metabolite profile. This phenomenon highlights the close correlation between the metabolic state of the fungus-infected host and its nutritional conditions. Collectively, our metabolomic findings provide a scientific basis for the quality evaluation of artificially reared BB. They also suggest its potential for yielding a distinct or broader spectrum of bioactive compounds, which could inform its future medicinal application.

## Figures and Tables

**Table 1:** Statistics of significant change DMs in AMF and MF groups.

Formula	Compounds	Class I	Log_2_FC	
			MF2 vs. MF1	AMF2 vs. AMF1
C_8_H_8_O_5_	3-O-Methylgallic acid	Phenolic acids	4.99	7.77
C_14_H_20_O_8_	5-(2-Hydroxyethyl)-2-O-glucosylphenol	Phenolic acids	−17.82	−7.01
C_17_H_22_O_10_	4-O-Glucosyl-sinapate	Phenolic acids	−1.56	−5.36
C_21_H_20_O_11_	Kaempferol-3-O-galactoside (Trifolin)	Flavonoids	−21.18	−22.02
C_14_H_17_N_5_O_8_	Succinyladenosine	Nucleotides and derivatives	−2.92	−2.39
C_23_H_48_NO_7_P	LysoPC 15:0 (2n isomer)	Lipids	−3.56	−5.17
C_24_H_48_NO_7_P	LysoPC 16:1	Lipids	−2.89	−4.75
C_26_H_46_NO_7_P	LysoPC 18:4	Lipids	−1.69	−3.19
C_27_H_50_NO_7_P	LysoPC 19:3	Lipids	−2.76	−2.56
C_28_H_48_NO_7_P	LysoPC 20:5	Lipids	−3.30	−3.95
C_28_H_36_O_13_	Syringaresinol-4′-O-g−lucoside	Lignans and Coumarins	−2.66	−3.82
C_4_H_6_O_4_	Methylmalonic acid	Organic acids	−2.16	−2.43
C_4_H_6_O_4_	Succinic acid	Organic acids	−2.16	−2.43
C_5_H_10_O_6_	D-Xylonic acid	Others	−1.06	−2.04
C_6_H_15_O_9_P	Sorbitol-6-phosphate	Others	−4.99	−6.93

Note: The extreme log_2_FC values present in the table are due to the fact that metabolites are undetectable in one group (the signal is at the background level) but are present in large quantities under other conditions. Therefore, the log_2_FC value represents a qualitative “on/off” switch rather than a quantifiable line change.

**Table 2:** Statistics of significant change DMs in AF group.

Formula	Compounds	Class I	Log_2_FC (AF2 vs. AF1)
C_14_H_20_N_6_O_5_S	S-(5′-Adenosy)-L-homocysteine	Amino acids and derivatives	1.57
C_10_H_13_N_5_O_3_	5′-Deoxyadenosine	Nucleotides and derivatives	15.76
C_6_H_5_N_5_O_2_	Isoxanthopterin	Nucleotides and derivatives	2.47
C_7_H_6_O_2_	4-Hydroxybenzaldehyde	Phenolic acids	−1.64
C_21_H_20_O_10_	Galangin-7-O-glucoside	Flavonoids	−3.72
C_15_H_10_O_6_	Kaempferol (3,5,7,4′-Tetrahydroxyflavone)	Flavonoids	−2.76
C_15_H_10_O_7_	Quercetin	Flavonoids	−2.61
C_15_H_10_O_7_	Morin	Flavonoids	−1.95
C_13_H_10_N_2_O	1-Acetyl-β-carboline	Alkaloids	−1.87
C_11_H_20_O_4_	Undecanedioic acid	Lipids	−1.74
C_18_H_30_O_4_	13(s)-hydroperoxy-(9z,11e,15z)-octadecatrienoic acid	Lipids	−1.23
C_8_H_16_NO_9_P	N-Acetyl-D-glucosamine-1-phosphate	Others	−2.03

**Table 3:** Statistical summary of pairwise comparison of DMs in the feeding groups (MF, AF, AMF) on D0 and D5.

Comparison	All Significant Difference	Relative Down	Relative Up
AMF1_vs_AF1	366	171	195
MF1_vs_AF1	337	191	146
MF1_vs_AMF1	264	185	79
AMF2_vs_AF2	301	209	92
MF2_vs_AF2	309	134	175
MF2_vs_AMF2	326	72	254

## Data Availability

All of the data used in this study have been provided in the main text and the Supplementary Materials.

## Supplementary Materials

The following supporting information can be downloaded at: https://www.mdpi.com/article/10.3390/metabo16010051/s1, Figure S1: Metabolic substance detection; Figure S2: Principal component analysis (PCA) plot; Figure S3: Analysis of differential metabolites; Figure S4: Statistics of metabolite changes in the three groups; Figure S5: Metabolite content difference analysis; Figure S6: Analysis of metabolite content differences; Table S1: PC1 and PC2 scores for each sample; Table S2: Fold change of metabolites during petrifaction; Table S3: Classification and group comparison of specific metabolites; Table S4: Between-group differences in metabolites.
