# Supplementary material for: Metabolomics Analysis Reveals the Potential Advantage of Artificial Diet-Fed Bombyx Batryticatus in Disease Treatment
Source: Metabolites. 2026 Jan 7;16(1):51. doi: 10.3390/metabo16010051 (PMC12843958; doi:10.3390/metabo16010051)
Supplement: Supplementary file 1 [file metabolites-16-00051-s001.zip › Supplementary figures.pdf]

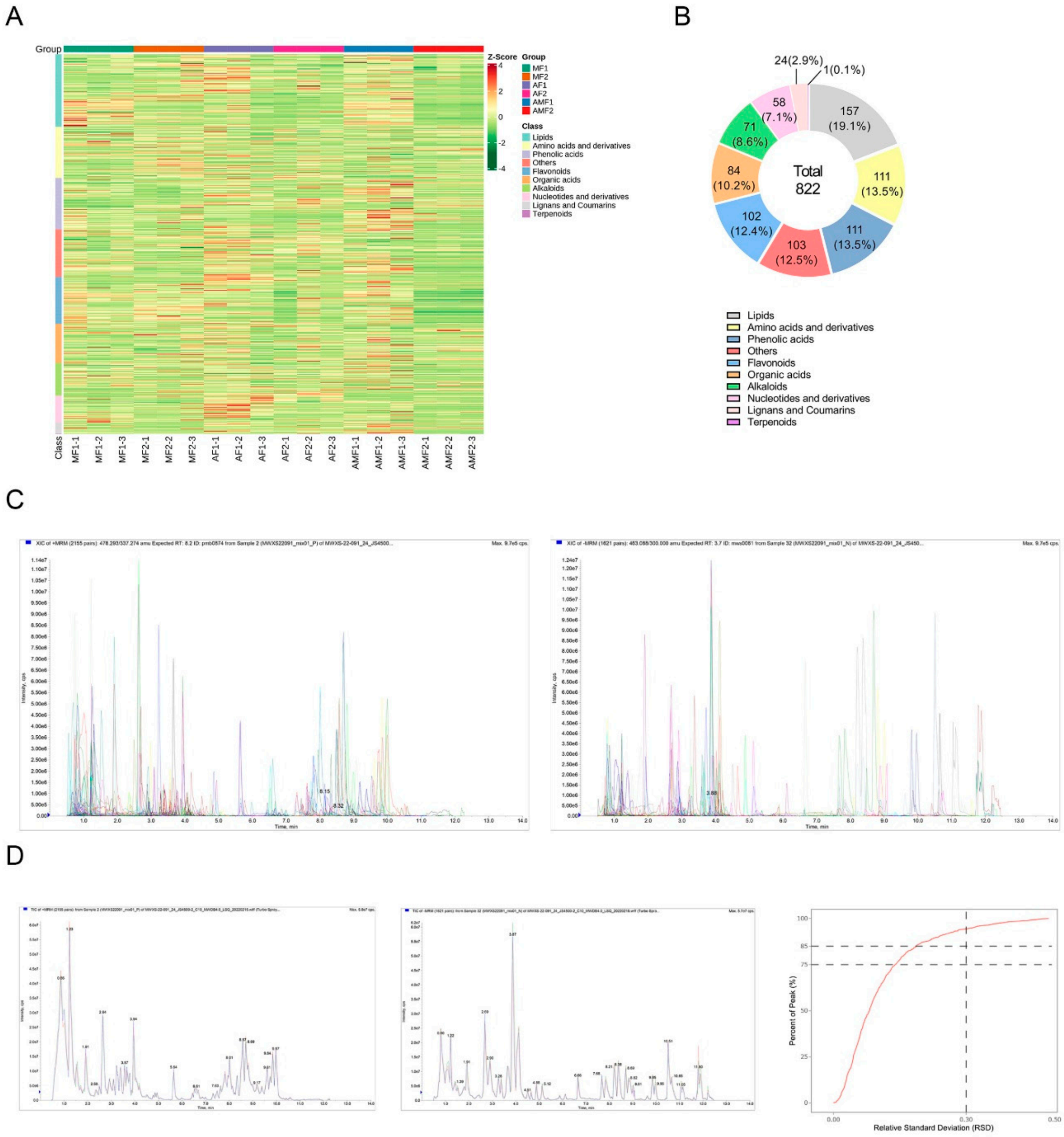

Figure S1. Metabolic substance detection. **(A)** Classification heat map of the 822 metabolites. **(B)** Classification and summary of 822 metabolites. **(C)** MRM Metabolite XICs. **(D)** QC sample mass spectrometry detection TIC overlap plot and RSD distribution plot.

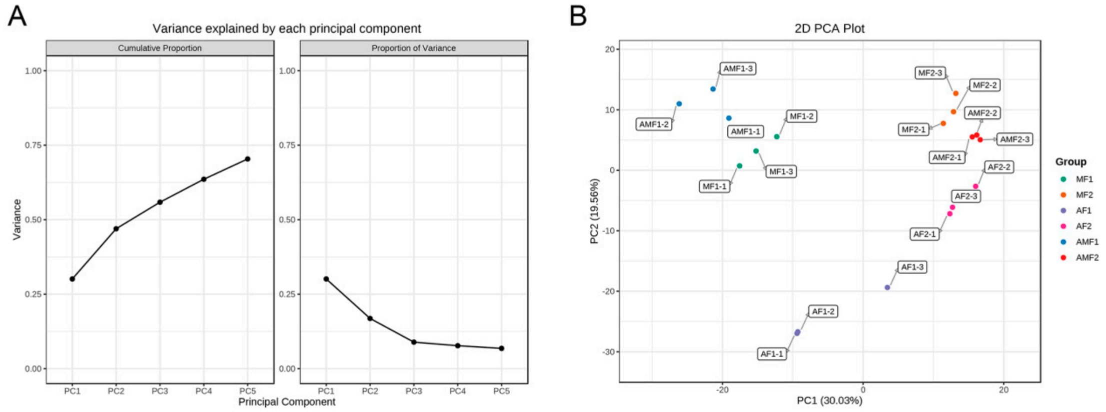

Figure S2. Principal component analysis (PCA) plot.

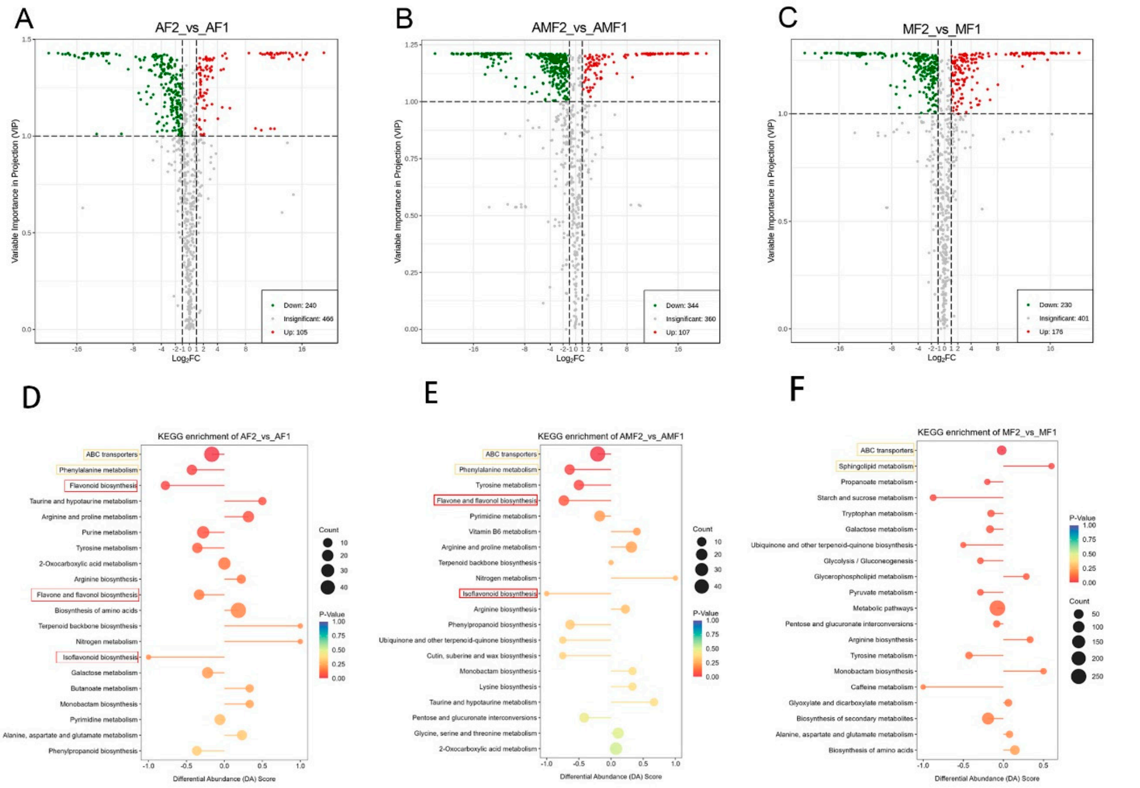

Figure S3. Analysis of differential metabolites. **(A-C)** Volcano diagram of DMs during the stiffening process, AF**(A)**, AMF**(B)**, MF**(C)**. **(D-F)** KEGG enrichment of DMs during the stiffening process, AF**(D)**, AMF**(E)**, MF**(F)**.

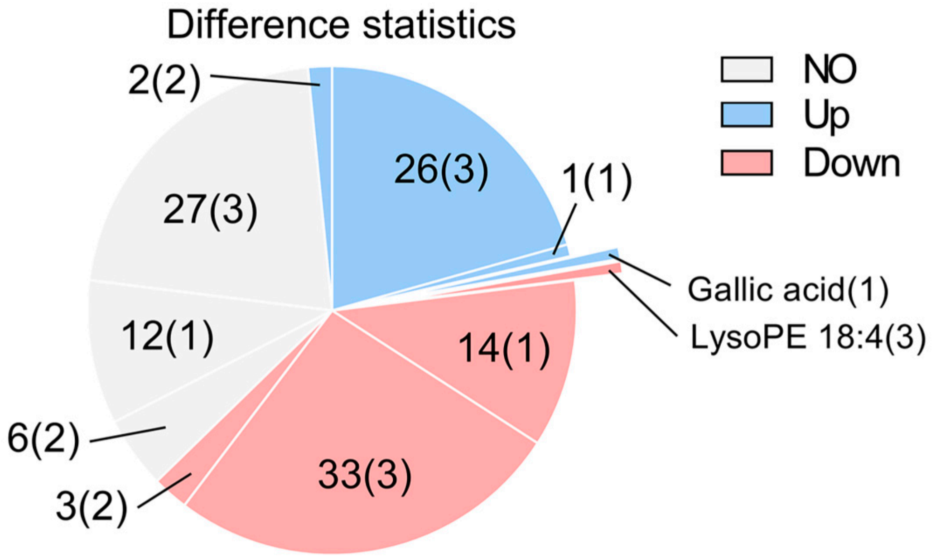

Figure S4: Statistics of metabolite changes in the three groups

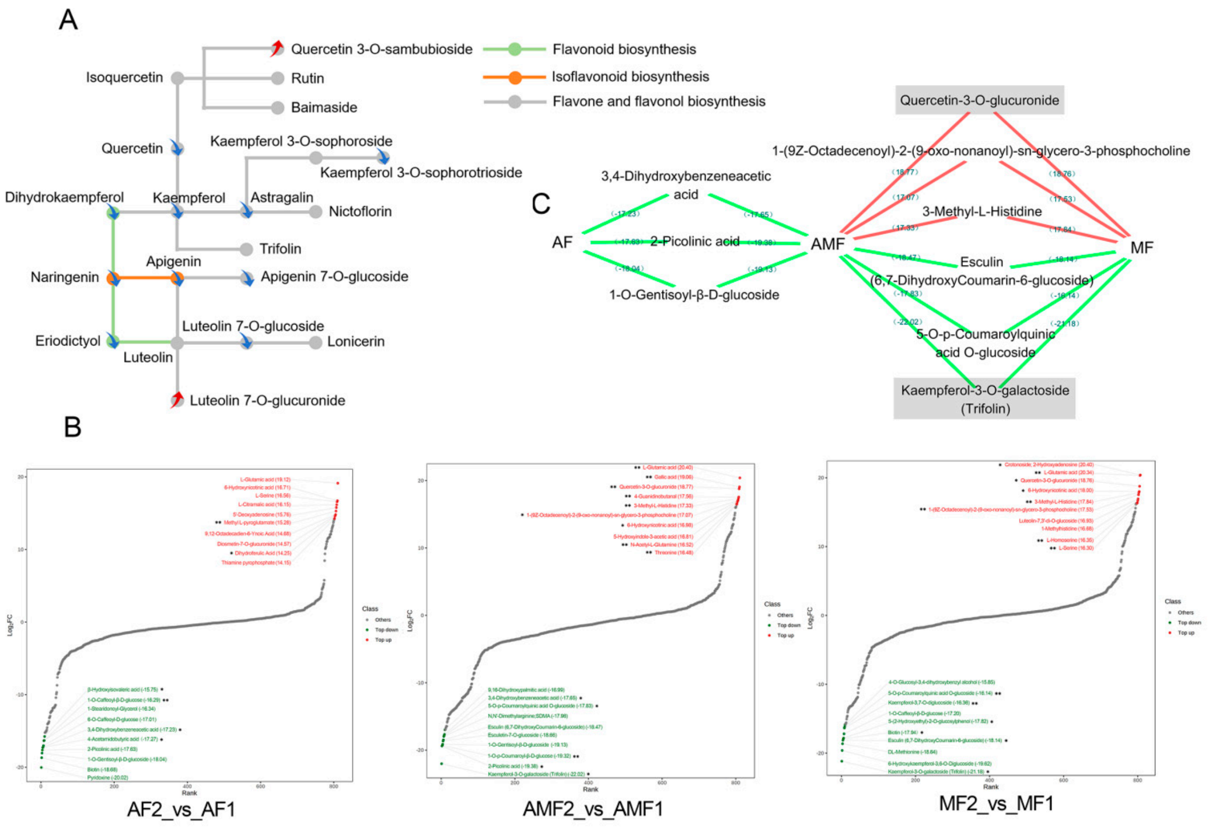

Figure S5. Metabolite content difference analysis. **(A)** Changes in flavonoids biosynthesis-related DMs in the AF group during the stiffening process. **(B)** The dynamic distribution of metabolite content in the three groups during stiffening process **(C)** Metabolite content analysis of the three groups during stiffening process.

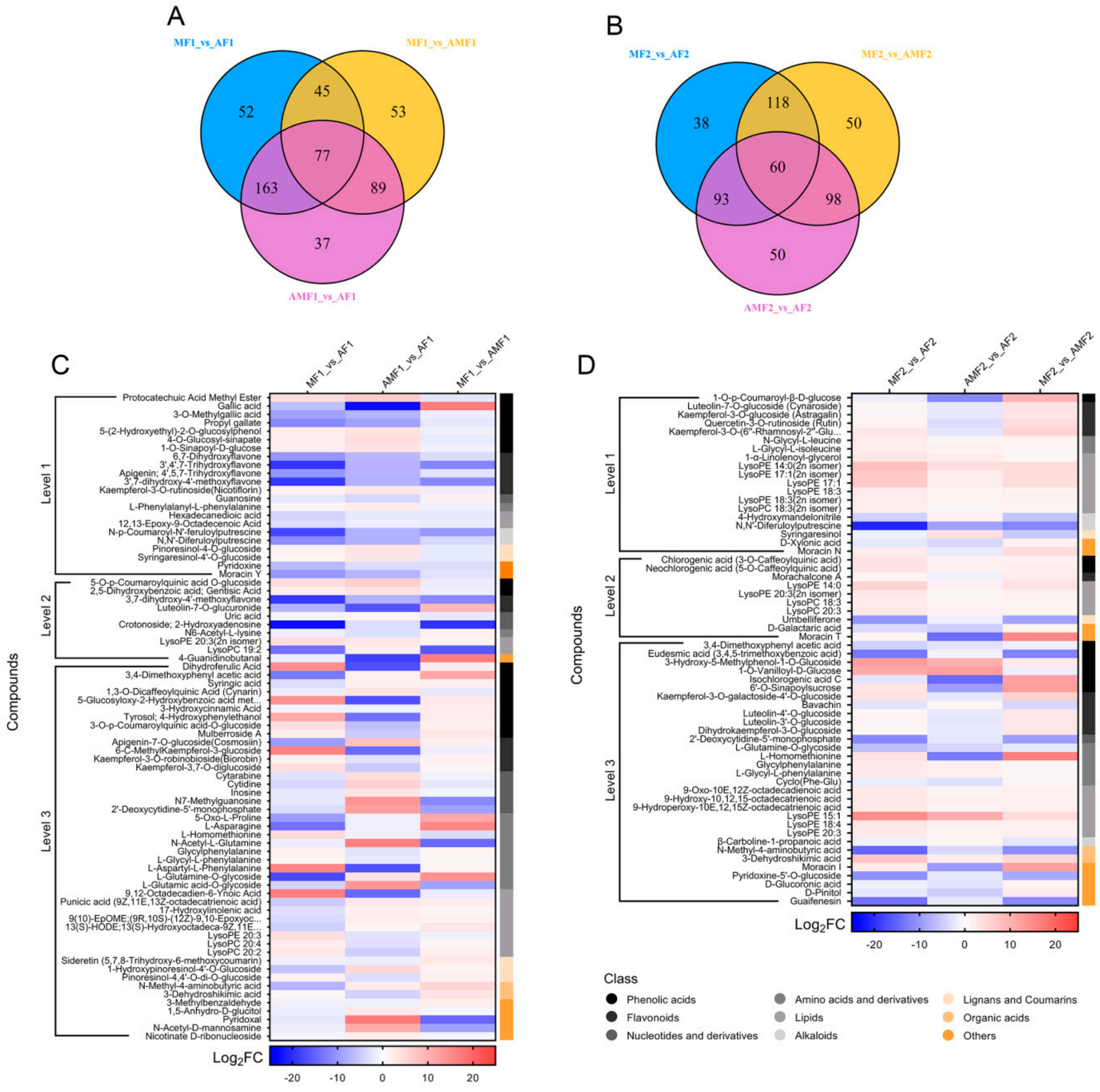

Figure S6. Analysis of metabolite content differences. **(A-B)** Venn diagrams of three groups of DMs between D0 and D5, D0 **(A)**, D5 **(B)**. **(C-D)** Heatmaps of DMs between D0 and D5 in the three groups, D0 **(C)**, D5 **(D).**
